# Genome-wide analysis of long non-coding RNAs at early stage of skin pigmentation in goats (Capra hircus)

**DOI:** 10.1186/s12864-016-2365-3

**Published:** 2016-01-19

**Authors:** Hangxing Ren, Gaofu Wang, Lei Chen, Jing Jiang, Liangjia Liu, Nianfu Li, Jinhong Zhao, Xiaoyan Sun, Peng Zhou

**Affiliations:** Chongqing Academy of Animal Sciences, Chongqing, 402460 China; Chongqing Engineering Research Center for Goats, Chongqing, 402460 China; Youyang Animal Husbandry Bureau, Chongqing, 409800 China

**Keywords:** Skin, lncRNA, Goat, Pigmentation, Transcriptome

## Abstract

**Background:**

Long noncoding RNAs (lncRNAs) play roles in almost all biological processes; however, their function and profile in skin development and pigmentation is less understood. In addition, because lncRNAs are species-specific, their function in goats has not been established.

**Result:**

We systematically identified lncRNAs in 100-day-old fetal skin by deep RNA-sequencing using the Youzhou dark goat (dark skin) and Yudong white goat (white skin) as a model of skin pigmentation. A total of 841,895,634 clean reads were obtained from six libraries (samples). We identified 1336 specific lncRNAs in fetal skin that belonged to three subtypes, including 999 intergenic lncRNAs (lincRNAs), 218 anti-sense lncRNAs, and 119 intronic lncRNAs. Our results demonstrated significant differences in gene architecture and expression among the three lncRNA subtypes, particularly in terms of density and position bias of transpose elements near the transcription start site. We also investigated the impact of lncRNAs on its target genes in *cis* and *trans*, indicating that these lncRNAs have a strict tissue specificity and functional conservation during skin development and pigmentation.

**Conclusion:**

The present study provides a resource for lncRNA studies in diseases involved in pigmentation and skin development. It expands our knowledge about lncRNA biology as well as contributes to the annotation of the goat genome.

**Electronic supplementary material:**

The online version of this article (doi:10.1186/s12864-016-2365-3) contains supplementary material, which is available to authorized users.

## Background

As a species of ubiquitous noncoding RNAs, long noncoding RNAs (lncRNAs) are unambiguously distinguished from mRNAs in terms of sequence structure, positional characteristics, expression level, and evolutional conservation [1–5]. Moreover, subspecies of lncRNAs have also been categorized and characterized in human [1, 4], zebrafish [5], and *Caenorhabditis elegans* [6]. Recent reports have shown that similar to mRNA, lncRNA is functional and spatiotemporally expressed in tissues [7–9]. Researchers have identified several functional lncRNAs associated with skin biology such as *ANCR*, *TINCR*, *U1 RNA*, *PRINS*, *BANCR*, and *SPRY4-IT1* [10]. In addition, it has also been shown that a few well-known oncogenes, including *H19*, *HOTTIP*, *Nespas*, *Kcnq1ot1*, *lincRNA-p21*, *mHOTAIR*, *Malat1*, *SRA*, *Foxn2-as*, *Gtl2-as*, and *H19-as*, are involved in vitamin D receptor protection against skin cancer formation by maintaining the balance of oncogenic to tumor-suppressing lncRNAs [11]. In current lncRNA databases, most of the identified lncRNAs were mainly derived from human and mouse [12–14]. Several recent studies in bovine [15–17], chicken [2], and pig [3, 18] have enriched the animal lncRNA datasets; however, our understanding of goat lncRNAs is limited. Despite the abundance of lncRNAs in the genome, only a few have been fully characterized. Currently, there are only two reports on the identification of the skin lncRNAs in mammals. RNA sequencing (RNA-seq) analysis conducted by Weikard et al. (2013) identified 4365 potential intergenic lncRNAs in cow with a piebald phenotype [17], which differs from that of Youzhou dark goat (as described in the next section). Another skin lncRNA catalog was derived from human skin cancer [11]. To our knowledge, only a few reports have described the involvement of skin lncRNAs in prenatal pigmentation and development. During embryonic development, fetal skin undergoes growth at a relatively high rate for 100 gestational days in goat [19]. Therefore, it is significant and necessary to investigate skin pigmentation during this specific developmental stage.

RNA-seq is a powerful approach that unravels the differential expression profiles underlying phenotypic differences, as well as deciphers non-annotated transcriptional activity by identifying various novel transcripts (protein-coding and noncoding) and additional alternative splice variants of known annotated transcripts [20–22]. In the current study, we elucidated the lncRNA profiles of two different phenotypes involved in skin pigmentation in goats using deep RNA-sequencing. Our study subject featured dark skin in its entire body, including its visible mucous membranes, and this phenotypic feature has not been reported in other mammals to date. Our study provides a valuable resource for studying lncRNAs that are involved in diseases, as well as contributes to better understanding the biology of skin pigmentation and development.

## Results

### Identification of lncRNAs in goat fetal skin

**Fig. 1:**
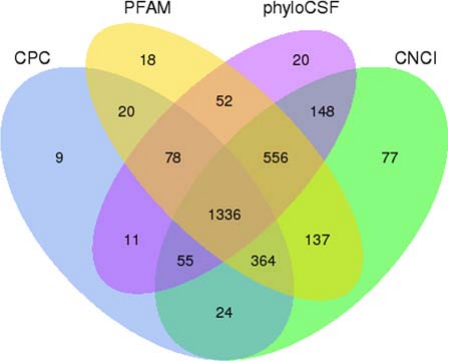
Screening of the candidate lncRNAs in skin transcriptome. Venn diagrams of coding potential analysis by using stringent criteria. Four tools (CPC, CNCI, PFAM, and PhyloCSF) were employed to analyze the coding potential of lncRNAs. Those simultaneously shared by four analytical tools were designated as candidate lncRNAs and used in subsequent analyses

**Fig. 2:**
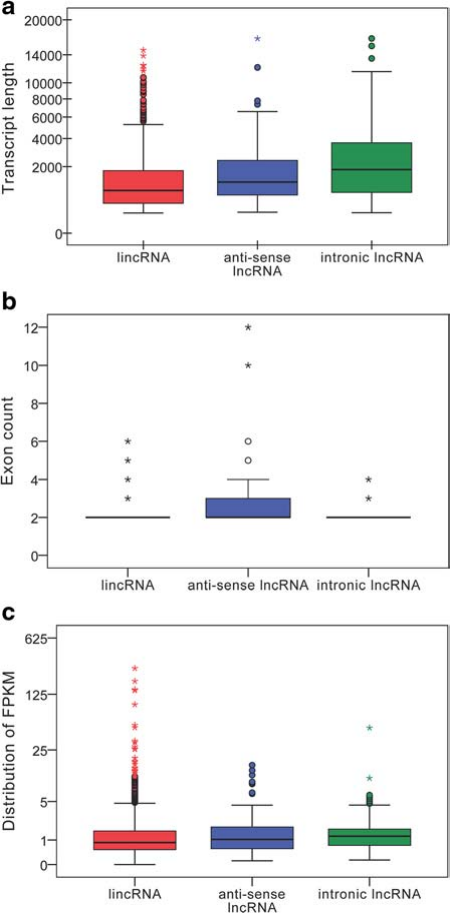
Comparative analysis of the three subtypes of lncRNAs. The transcript length (**a**), exon count (**b**), and expression level (**c**) of three subtypes of lncRNAs were compared using the Kolmogorov-Smirnov test, and a *P* value of 0.05 indicates significance between two groups. In three box plots, the circle indicates the outlier, and the asterisk labels the extreme

A total of 923,013,870 raw reads were produced from the Illumina HiSeq 2000 platform. After discarding adaptor sequences and low-quality sequences, we obtained 841,895,634 clean reads (accounting for 84.2 Gb), and the percentage of clean reads among raw tags in each library ranged from 88.39–93.02 % (Additional file 1). Subsequently, we mapped the clean reads based on the latest goat reference genome (http://goat.kiz.ac.cn). Considering the characteristics of lncRNA sequences (≥200 nt, exon count ≥ 2) and its differences from other classes of RNA (mRNA, tRNA, rRNA, snRNA, snoRNA, pre-miRNA, and pseudogenes), we classified the transcripts into different subtypes using both Scripture (beta2) and Cufflinks (v2.1.1). Our results showed that 93.6 % of the 46,933 identified transcripts were known reference transcripts, whereas 6.29 % (2952) were the presumed lncRNAs. To further confirm these 2952 lncRNAs, we performed coding potential analysis using the software CNCI, CPC, Pfam-scan, and PhyloCSF. After screening using rigorous criteria and four analytic tools, a total of 1336 lncRNAs from the skin of fetal goats were identified and subjected to further analysis (Fig. 1). The 1336 lncRNAs consisted of 999 large intergenic noncoding RNAs (lincRNAs), 218 intronic_lncRNAs, and 119 anti-sense_lncRNAs. A preliminary analysis revealed major differences in gene architecture and expression levels among the three subtypes of lncRNAs. For example, the length of intronic lncRNAs was longer than that of lincRNAs (Kolmogorov-Smirnov test, *P* = 0.000) and anti-sense lncRNAs (Kolmogorov-Smirnov test, *P* = 0.005), with a median length of 1.831 kb *vs*. 0.842 kb and 1.194 kb, respectively. Significant differences in transcript length between lincRNAs and intronic lncRNAs were also observed (Kolmogorov-Smirnov test, *P* = 0.001; Fig. 2a). On the other hand, clear differences in the number of exons were also observed among the three lncRNA subtypes (Fig. 2b). In particular, the anti-sense lncRNAs showed a higher number of exons and wider size distribution than that observed in the lincRNAs (Kolmogorov-Smirnov test, *P* = 0.222) and intronic lncRNAs (Kolmogorov-Smirnov test, *P* = 0.001). We also detected significant differences in exon distribution between lincRNAs and intronic lncRNAs (Kolmogorov-Smirnov test, *P* = 0.016). In terms of expression level based on fragments per kb for a million reads (FPKM) values, the intronic lncRNAs showed a higher expression level than that of the lincRNAs (Kolmogorov-Smirnov test, *P* = 0.000) and anti-sense lncRNAs (Kolmogorov-Smirnov test, *P* = 0.037), with a median of 1.229 *vs*. 0.8607 and 1.035, respectively (Fig. 2c). The diversity in gene architecture and expression levels among various types of lncRNAs may have implications in its specific function in the goat genome.

### Transposable elements characterize various subtypes of lncRNAs

**Fig. 3:**
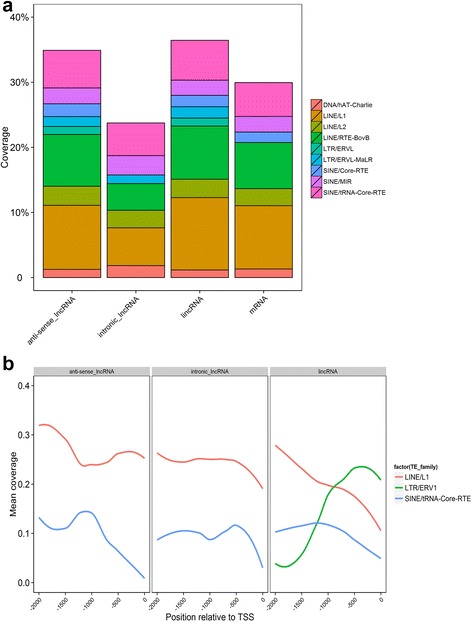
TE components and position bias in three subtypes of goat lncRNAs. The main TE families were identified using RepeatMasker in the 999 lincRNAs, 218 intronic_lncRNAs, 119 anti-sense_lncRNAs, and 27,947 mRNAs, respectively. Differences in TE components between mRNAs and individual subtype of lncRNAs were measured by using the Fisher Exact test (**a**). To ascertain the position bias of TE components in three subtypes of lncRNAs, we identified TEs approaching TSSs in three classes of lncRNAs in the goat genome (http://goat.kiz.ac.cn) and plotted the coverage of various TE families (**b**)

Transposable elements (TEs) are mobile genetic elements that are capable of movement and proliferation within the genome, and its remnants account for one to two thirds of mammalian genomes [23, 24]. TEs are also considered as one of three evolutionary scenarios involved in the origin of lncRNAs [25]. We were thus prompted to identify differences in TE components between lncRNAs and mRNAs, as well as among the three lncRNA subtypes. Our analysis revealed TE component characteristics that distinguished the three lncRNA subtypes (Fig. 3a). At the global level, significant differences were observed between mRNAs and the individual subtype of lncRNAs (Additional file 2). Among the three subspecies of lncRNAs, the intronic lncRNAs showed a lower TE density than that observed in the lincRNAs and anti-sense lncRNAs (23.75 % *vs*. 36.44 % and 34.91 %). In particular, the lincRNAs and anti-sense lncRNAs have a relatively higher proportion of LINEs/L1s (Fisher’s Exact, *P*_linc *vs.* intronic_ = 0.0034; *P*_anti-sense *vs*. intronic_ = 0.0011) and LINEs/RTE-BovBs (Fisher’s Exact, *P*_linc *vs*. intronic_ = 5.55E-05; *P*_anti-sense *vs*. intronic_ = 0.0024) than that observed in intronic lncRNAs. In contrast to the lincRNAs and anti-sense lncRNAs, the intronic lncRNAs showed a deletion of LTRs/ERVLs (Fisher’s Exact, *P*_linc *vs.* intronic_ = 5.79E-08; *P*_anti-sense *vs*. intronic_ = 2.11E-05) and SINEs/Core-RTEs (Fisher’s Exact, *P*_linc *vs*. intronic_ = 2.38E-05; *P*_anti-sense *vs.* intronic_ = 0.0005). Differences in the density of other TE subspecies were observed among the three lncRNA subtypes (Additional file 3). These structural characteristics of TE components may underlie the differences in the evolution of the three lncRNA subtypes. Long terminal repeats (LTRs) harbor promoter signals that modulate gene expression in genomes [26, 27], and a recent study has indicated that endogenous retroviruses (ERVs), which is a class of LTRs, exhibit position and orientation biases, often preferring the 5′ end of lincRNA transcripts and sense orientation within the transcript, and avoiding the mRNA transcription start sites (TSSs) [28]. We were thus prompted to identify position bias for LTRs relative to TSSs among the three lncRNA subtypes. The LTR/ERV1 showed a large coverage peak right at the TSS of lincRNAs, whereas a deletion of the LTR/ERV1 was observed in the anti-sense lncRNAs and intronic lncRNAs (Fig. 3b). Furthermore, the anti-sense lncRNAs and intronic lncRNAs also exhibited a relatively higher coverage of LINEs/L1s at its TSSs. These findings were suggestive of differential mechanisms of transcription regulation among the lincRNAs and the other two lncRNA subtypes.

### Comparison of features of mRNAs and lncRNAs

**Fig. 4:**
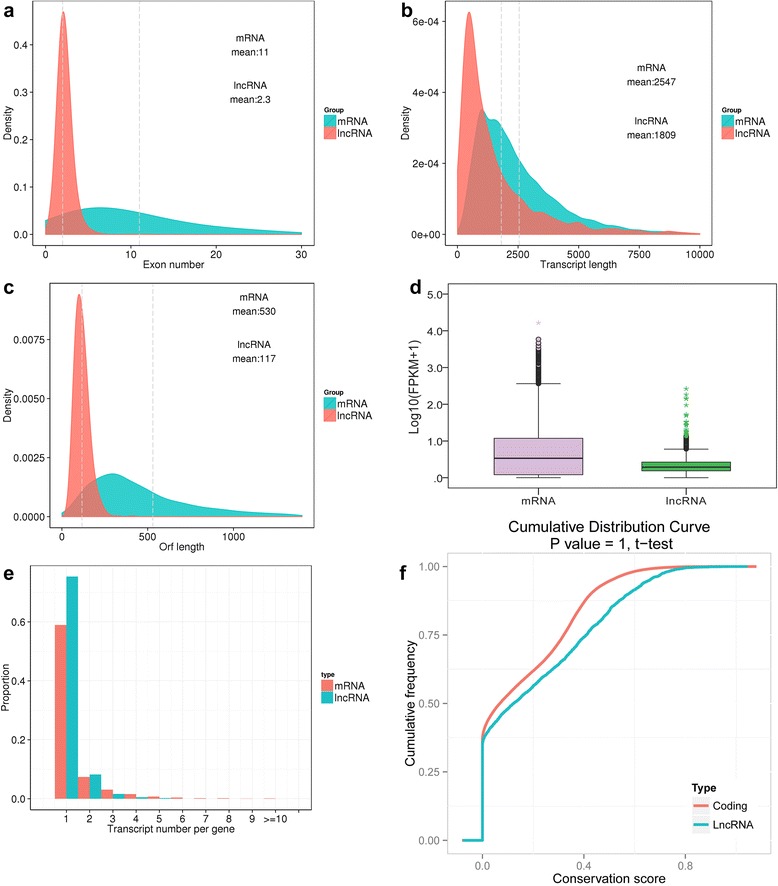
Comparison of genomic architecture and expression level between mRNAs and lncRNAs. The mRNAs and lncRNAs identified in the present study were used for comparison of primary differences in two classes of transcripts. **a** Distribution of transcript lengths in the mRNAs and lncRNAs in skin. The horizontal axis of indicates the length of transcripts, and the vertical axis represents density. **b** Distribution of the number of exons in the mRNAs and lncRNAs. In the present study, single-exon lncRNAs were filtered out from the goat genome due to the limitations of the algorithm. **c** Distribution of the number of open reading frames (Orfs) in the mRNAs and lncRNAs. The Orf was identified using Estscan in the present study. **d** Expression level indicated by log_10_(FPKM + 1) in the mRNAs and lncRNAs. **e** Proportional distribution of alternative splicing transcripts in mRNAs and lncRNAs. **f** Conservation of the sequence in mRNAs and lncRNAs were evaluated using phastCons (http://compgen.bscb.cornell.edu/phast/)

**Fig. 5:**
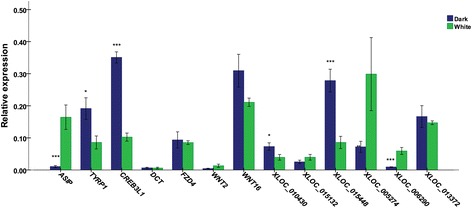
Validation of gene expression in dark and white skin by quantitative PCR. Some identified melanogenic genes and lncRNAs were examined in dark and white skin of fetal goats using quantitative PCR. Gene expression was quantified relative to the expression level of *β*-*actin* using the comparative cycle threshold (∆CT) method. Correction for multiple comparisons was performed using the Holm-Sidak method. The data are expressed as the mean ± 1 SE (*n* = 3). * *P* < 0.05, **P < 0.01, *** *P* < 0.001

In the present study, we obtained a total of 27,947 mRNAs and 1336 lncRNAs from goat fetal skin. To comprehensively examine the differences between the two transcript species, comparative analysis of gene structure, expression, and sequence conservation was performed. Our results showed that 1) most of lncRNAs contained two or three exons, which differs from that of mRNAs (Fig. 4a); 2) there was a distinct divergence in the distribution of transcript length between mRNAs and lncRNAs (Fig. 4b); 3) most of the lncRNAs contained a relatively shorter ORF, compared to that of mRNAs (Fig. 4c); 4) lncRNAs generally showed a lower level of expression compared to that observed in mRNAs (Fig. 4d); 5) lncRNAs often generate a lower number of alternatively spliced transcripts, in contrast to that in mRNAs (Fig. 4e); and 6) most lncRNAs are slightly less conserved, although not statistically significant (Fig. 4f).

### The *cis* and *trans* role of lncRNAs in target genes

To investigate the function of lncRNAs, we predicted the potential targets of lncRNAs in *cis* and *trans*. For the *cis* action of lncRNAs, we searched for protein-coding genes 10 and 100 kb upstream and downstream of the lncRNAs, respectively. Our results included 641 lncRNAs that corresponded to 868 protein-coding genes within a range of 10 kb, as well as 964 lncRNAs that represented 3468 protein-coding genes within a range of 100 kb (Additional file 4). Interestingly, we detected melanogenic genes such as *ASIP*, *Mitf*, *Sox10*, *Wnt7b*, and *Wnt3a*, which were respectively located near the *XLOC_005274*, *XLOC_013722*, *XLOC_020482*, *XLOC_020548*, and *XLOC_022579* loci, thereby suggesting that skin melanogenesis is regulated by the action of five lncRNAs on neighboring protein-coding genes. Gene Ontology (GO) analysis of *cis* lncRNA targets demonstrated that 25 significantly overrepresented terms were mainly involved in the regulation of gene expression. For example, the top five terms were sequence-specific DNA binding, nucleic acid binding transcription factor activity, sequence-specific DNA binding transcription factor activity, regulation of transcription, and DNA-dependent regulation of RNA metabolic processes. These findings clearly demonstrated one of the roles of lncRNAs in the genome, namely, regulation of gene expression. Pathway analysis showed that these *cis* target genes of lncRNAs were enriched in 266 KEGG pathways, in which several pathways were related to pigmentation such as tyrosine metabolism, cAMP signaling pathway, MAPK signaling pathway, Wnt signaling pathway, melanogenesis, and melanoma (Additional file 5). These findings suggested that lncRNAs act on its neighboring protein-coding genes in *cis* to regulate skin pigmentation during dermal development.

On the other hand, the *trans* role of 1336 lncRNAs in protein-coding genes was examined based on its expression correlation coefficient (Pearson correlation ≥ 0.95 or ≤ −0.95). A total of 123,969 interaction relationships were detected in *trans* between 1150 lncRNAs and the protein-coding genes in the goat genome (Additional file 6). Functional analysis illustrated that the *trans* target genes were enriched in 2643 GO terms, encompassing a variety of biological processes. Importantly, we observed a few of melanogenic terms, including pigment biosynthetic process, tyrosine 3-monooxygenase activity, melanin-concentrating hormone activity, pigment metabolic process, nitrogen compound metabolic process, and others. Of the 256 KEGG pathways identified, five were associated with pigmentation such as melanogenesis, melanoma, Wnt signaling pathway, cAMP signaling pathway, and tyrosine metabolism (Additional file 7). These findings indicated that lncRNAs act on the protein-coding genes associated with skin pigmentation in *trans*.

To further ascertain lncRNA-protein-coding gene pairs that belong to both co-localization (*cis* action) and expression correlation (*trans* action) relationships, detailed examination was conducted, which identified 26 lncRNA-protein coding gene pairs that fulfilled to these criteria (Additional file 8). This finding suggested that lncRNAs act on its neighboring protein-coding genes to regulate gene expression. We also noticed that one lncRNA, *XLOC_020022*, which was significantly differentially expressed between goats with dark skin and white skin, interacted with an early development-related gene, *HOXC11*.

### Tissue and functional specificities of lncRNAs

Expression correlation analysis revealed an interesting phenomenon wherein an lncRNA in *trans* acted on two protein-coding genes that were specifically expressed in a particular type of cell or belonged to a certain functional cluster. For example, *XLOC_013372* targets *ASIP* and *MITF*, yet with opposite correlations. A group of lncRNAs, including *XLOC_023806*, *XLOC_019686*, *XLOC_008226*, *XLOC_013939*, *XLOC_015399*, *XLOC_017870*, *XLOC_000404*, and *XLOC_002582* simultaneously act on both *TYRP1* and *DCT*; *XLOC_010430*, *XLOC_000995*, *XLOC_019547*, *XLOC_009688*, *XLOC_005961*, and *XLOC_006605* target both *WNT2* and *CREB3L1*; and lncRNAs target *WNT2* and *FZD4*, respectively. On the other hand, *ASIP*, *MITF*, *TYRP1*, and *DCT* are members of melanogenic pathways and are expressed specifically in melanocytes. These findings indicate that the lncRNAs are tissue- or function-specific. Furthermore, the three unique differentially expressed lncRNAs (*XLOC_010430*, *XLOC_004341*, and *XLOC_015448*) between the normal and dark skin in goats require further investigation because their targets were also differentially expressed, except for *MITF* (the target of *XLOC_015448*). We suspect that these lncRNAs most probably participated in the regulation of melanogenesis, although its underlying mechanisms require additional investigations. Selected lncRNAs and target genes related to pigmentation were validated by quantitative PCR analysis (Fig. 5, Table 1).

**Table 1:** LncRNAs and its potential target genes that are involved in melonagenesis

Protein-coding genes	lncRNAs in *cis*	lncRNAs in *trans*
*ASIP* ^a^	XLOC_005274	*XLOC_013372*, XLOC_006290, XLOC_013615, XLOC_024549,
	XLOC_004858	XLOC_000129, XLOC_006932, XLOC_020962, -XLOC_020019^b^, -XLOC_018830^b^
*MITF*	XLOC_013722	*-XLOC_013372* ^b^, -XLOC_022057^b^, XLOC_000689, XLOC_018361, XLOC_012968, XLOC_009509, XLOC_023640, -XLOC_013890^b^, XLOC_005088, XLOC_015448^a^
*TYRP1* ^a^ (*BROWN*)		*XLOC_023806*, *XLOC_019686*, *XLOC_008226*, *XLOC_013939*, *XLOC_015399*, *XLOC_017870*, *XLOC_000404*, *XLOC_002582*
*DCT* ^a^		*XLOC_023806*, *XLOC_019686*, *XLOC_008226*, *XLOC_013939*, *XLOC_015399*, *XLOC_017870*, *XLOC_000404*, *XLOC_002582*, -XLOC_005274^b^
*TYR*		-XLOC_010559^b^, -XLOC_024478^b^, XLOC_021855, XLOC_006064
*CREB3L1* ^a^		*XLOC_000995*, *-XLOC_009688* ^b^, *XLOC_005961*, *XLOC_006605*, *XLOC_004319*, *XLOC_008730*, *XLOC_010430* ^a^, XLOC_019547, -XLOC_000912^b^, XLOC_024598, XLOC_004263
*FZD4* ^a^		*XLOC_000995*, *XLOC_018035*, *XLOC_005957*, *XLOC_006451*, *XLOC_004319*, *XLOC_020603*, *XLOC_009285*, *XLOC_019333*, *XLOC_004805*, *XLOC_008730*, *XLOC_010430* ^a^, *XLOC_006605*, *XLOC_003840*, XLOC_002867^a^, XLOC_023214, XLOC_023692, XLOC_002389^a^
*WNT2* ^a^		*XLOC_000995*, *XLOC_018035*, *XLOC_005957*, *XLOC_006451*, *XLOC_004319*, *XLOC_020603*, *XLOC_009285*, *XLOC_019333*, *XLOC_004805*, *XLOC_008730*, *XLOC_010430* ^a^, *XLOC_006605*, *XLOC_003840*, *XLOC_019547*, *-XLOC_009688* ^b^, *XLOC_005961*, XLOC_004341^a^, -XLOC_014182^b^, XLOC_013150, XLOC_022462, XLOC_013012, XLOC_025297^a^, XLOC_008538, -XLOC_007438^b^, XLOC_005975, XLOC_004597

^a^Differentially expressed in dark and normal skins of goats

^b^Negative correlation between the lncRNAs and their targets in trans. Italic font indicates that one lncRNA acts on at least two different protein-coding genes in *trans*. For example, *XLOC_013372* regulated *ASIP* and *MITF* in *trans*

## Discussion

In the present study, we identified a total of 1336 multiple-exon lncRNAs in 100-day-old fetal goat skin. In contrast to the number of protein-coding genes identified in the present study (27,947 mRNAs), the expression of lncRNAs was tissue-specific [1]. Comparative analysis of lncRNAs and mRNAs revealed characteristics that were similar to those of recent studies [1–5]. In addition to the preliminary examination of the three lncRNA subtypes, our extensive characterization revealed major differences in TE components (LINEs/L1s, LINEs/RTE-BovBs, LTRs/ERVLs, and SINEs/Core-RTEs) among lincRNAs, intronic lncRNAs, and anti-sense lncRNAs (Fig. 3a), which may in turn be responsible for the observed differences in their evolution and function. Because about half of mammalian genomes consist of lncRNAs [25], our results might provide insights into the scenario of genome evolution via lncRNA evolution. LTRs are known to harbor promoter signals that modulate gene expression in genomes [26, 27]. Our findings have demonstrated that lincRNAs are highly enriched with LTRs/ERV1 at TSSs, but absent in anti-sense lncRNAs and intronic lncRNAs (Fig. 3b), which suggest that the regulatory mechanism of expression of lincRNAs differs from that of the other two subtypes. A recent study showed distinct differences in TE density and position bias between the lincRNAs and mRNAs [28], whereas the present study improves our understanding of lncRNA biology.

In 2013, the goat genome was sequenced and assembled *de novo* using whole-genome mapping technology [29], which endows its high quality of genome assembly and annotation among farm animals, including horse, pig, cattle, yak, and sheep [30–34]. Although some single-exon lncRNAs were filtered out of the goat genome due to the limitations of the algorithm of the present study, authentic multiple-exon lncRNAs were generated, which could then be utilized in future investigations, as well as considerably improve the annotation of the goat genome. On the other hand, unlike protein-coding genes where sequence motifs are usually indicative of function, lncRNA sequences are currently uninformative for predicting function. This particular limitation hinders the prediction of the function of lncRNAs. Interestingly, several lncRNAs are transcribed with their associated protein-coding transcripts [35], and various examples of recently characterized ncRNAs such as Evf2 [36], HOTAIR [37], Kcnq1ot1 [38], and Air [39] support a functional relationship between lncRNAs and its associated or related-protein coding gene(s). Therefore, functional predictions for mammalian lncRNAs have often been based on “guilt-by-association” analyses [1, 5, 40–42], although this may not be the most appropriate model to explain the function of lncRNAs.

We predicted the potential function of lncRNAs in goat skin and determined that protein-coding genes can act with lncRNAs in *cis* or *trans*. In particular, *ASIP*, which was most differentially expressed in dark and white skin as indicated by RNA-seq analysis, was determined to be regulated by several lncRNAs both in *cis* and in *trans* (Table 1). However, the mechanism by which the lncRNAs act on *ASIP* in *cis* and *trans* remains to be elucidated. An intriguing observation is that *XLOC_013372* acts on both *ASIP* and *MITF* in *trans*, but with reverse correlations (Table 1). This is the first report of such an interesting observation, which is worth investigating further, as well as indicates the functional complexity of lncRNAs. Furthermore, a certain cluster of lncRNAs in *trans* often target protein-coding genes that were specifically expressed in melanocytes (*ASIP*, *MITF*, *TYPR1*, and *DCT*) and/or involved in melanogenesis (*WNT2*, *WNT16*, *FZD4*, and *CREB3L1*) (Table 1). This finding indicates that lncRNAs play a regulatory role in melanogenesis. Moreover, a cluster of eight lncRNAs act on both *TYRP1* and *DCT*, which evolved from a common ancestral tyrosinase gene [43–45]. The observation of highly identical regulatory lncRNAs suggests that these homologous sequences in the tyrosinase family genes are involved in its evolution and functionality. A third interesting observation is that *FZD4* and *WNT2*, which are members of the WNT signaling pathway, share a few of regulatory lncRNAs, including significantly differentially expressed *XLOC_010430*. This again indicates that lncRNAs are highly functionally conserved, similar to their targets, namely, the WNT signaling proteins [46]. Several recent studies also indicate that lncRNAs are conserved in function [41, 47, 48]. Functional conservation, despite variations in sequence, is a characteristic of lncRNAs. The differentially expressed lncRNAs between dark and white skin in goats such as *XLOC_015448*, *XLOC_002867*, *XLOC_002389*, *XLOC_010430*, *XLOC_004341*, and *XLOC_025297* (Table 1), as well as the 26 lncRNA-protein coding gene pairs that belong to both co-localization (*cis* role) and correlation (*trans* role) (Additional file 8) require additional investigations.

As far as we know, only a small portion of the pathways involved in pigmentation have been validated to date, including the protein kinase C pathway [49, 50], cAMP pathway [51], SCF-KIT pathway [52], cGMP pathway [53], phosphatidylinositol 3-kinase-Akt pathway [54], protein kinase A pathway [55], BMP signaling [56], Notch pathway [57], ERK pathway [58], Wnt signal [59], KITLG and the KITLG/c-Kit pathway [60], CXCR3-mediated pathway [61], JAK2-STAT6 signaling pathway [62], nitric oxide/protein kinase G signaling pathway [63], FGF/MAPK/Ets signaling [64], p38MAPK [65], MITF-GPNMB pathway [66], Galphas-SASH1-IQGAP1-E-cadherin pathway [67], CREB/MITF/tyrosinase pathway [68], and necrosis factor receptor-associated factor 2 (TRAF2)-caspases pathway [69]. However, reports on the role of lncRNA in pigmentation are limited. In the present study, the enriched KEGG pathways associated with pigmentation (Additional files 5 and 7) in the potential lncRNA targets clearly indicated that these lncRNAs play roles in skin pigmentation in goats. However, the predicted targets based on “guilt-by-association” analyses should be carefully assessed because of the low number of sample examined, and experimental validations are also warranted.

## Conclusions

We elucidated the skin lncRNA profiles of fetal goats using deep RNA-seq analysis. The characterization of three lncRNA subtypes casts light on the mechanism underlying the origin and evolution of lncRNAs, as well as its regulation of expression. LncRNAs are tissue-specific and functionally conserved during skin development and pigmentation in goats. Our findings have further expanded our knowledge on lncRNA biology, as well as contributed to the annotation of the goat genome. The present study also provides valuable resources for studying lncRNAs.

## Methods

### Animals

Two goat groups with diverse phenotypes of skin pigmentation were investigated in this study. The Yudong white goat (*Capra hircus*) is distributed in Southwest China (located at 31°14′–32°12′ N and 108°15′–109°58′ E), which features white color coat and skin. The Youzhou dark goat (*Capra hircus*), a indigenous breed uniquely distributed in Youyang county in Chongqing, China (located at 26°54′ N and 108°57′ E), is characterized by dark skin, including the visible mucous membranes, yet is generally white in coat color. Briefly, three pregnant ewes from each breed were subjected to caesarean section to collect the fetuses at 100 days of gestation, and then the dorsal and ventral skins were collected from each fetus. Three grams of skin were dissected and rapidly frozen in liquid nitrogen for RNA extraction.

All surgical procedures involving goats were performed according to the Regulations for the Administration of Affairs Concerning Experimental Animals (Ministry of Science and Technology, China; revised in June 2004) and adhered to the Reporting Guidelines for Randomized Controlled Trials in Livestock and Food Safety (REFLECT).

### RNA isolation, library preparation, and sequencing

Total RNA was isolated using TRIzol reagent (Invitrogen, Carlsbad, CA, USA), according to the manufacturer’s instructions. RNA degradation and contamination was monitored on 1 % agarose gels. RNA purity was checked using the NanoPhotometer spectrophotometer (Implen, Los Angeles, CA, USA). RNA concentration was measured using a Qubit RNA Assay Kit in a Qubit 2.0 Fluorometer (Life Technologies, Carlsbad, CA, USA). RNA integrity was assessed using the RNA Nano 6000 Assay Kit of the Bioanalyzer 2100 system (Agilent Technologies, Santa Clara, CA, USA). Approximately 3 μg RNA per sample was used as input material for the RNA sample preparations. First, ribosomal RNA was removed by using an Epicentre Ribo-zero rRNA Removal Kit (Epicentre, Madison, WI, USA), and rRNA-free residue was removed by ethanol precipitation. Subsequently, the high strand-specificity of the libraries was generated using the rRNA-depleted RNA of the NEBNext Ultra Directional RNA Library Prep Kit for Illumina (NEB, Ipswich, MA, USA), following manufacturer’s recommendations. Briefly, fragmentation was conducted using divalent cations under elevated temperature in NEBNext. First-strand cDNA was synthesized using random hexamer primers and M-MuLV Reverse Transcriptase (RNaseH-). Second-strand cDNA synthesis was subsequently performed using DNA Polymerase I and RNase H. In the reaction buffer, dNTPs with dTTP were replaced by dUTP. Remaining overhangs were converted into blunt ends via exonuclease/polymerase activities. After adenylation of the 3′ ends of the DNA fragments, NEBNext adaptors with a hairpin loop structure were ligated to prepare for hybridization. To preferentially select cDNA fragments of 150–200 bp in length, the library fragments were purified with an AMPure XP system (Beckman Coulter, Brea, CA, USA). Then 3 μL USER Enzyme (NEB, Ipswich, MA, USA) was used with size-selected, adaptor-ligated cDNA at 37 °C for 15 min followed by 5 min at 95 °C before PCR. Then, PCR was performed with Phusion High-Fidelity DNA polymerase, universal PCR primers, and Index (X) Primers. Finally, the PCR products were purified (AMPure XP system), and library quality was assessed on an Agilent Bioanalyzer 2100 system. Clustering of the index-coded samples was performed on a cBot Cluster Generation System using TruSeq PE Cluster Kit v3-cBot-HS (Illumina, San Diego, CA, USA), following the manufacturer’s instructions. After cluster generation, the libraries were sequenced on an Illumina Hiseq 2000 platform and 100-bp paired-end reads were generated.

### Quality control

Raw data were first processed using in-house Perl scripts. In this step, clean data were obtained by removing reads containing adapter, reads containing over 10 % of ploy-N, and low-quality reads (>50 % of bases whose Phred scores were <5 %) from the raw data. The Phred score (Q20, Q30) and GC content of the clean data were calculated. All subsequent analyses were based on the high-quality data.

### Transcriptome assembly

Goat reference genome and gene model annotation files were downloaded from the goat genome website (http://goat.kiz.ac.cn) directly. Index of the reference genome was built using Bowtie v2.0.6 [70, 71] and paired-end clean reads were aligned to the reference genome using TopHat v2.0.9 [72, 73]. The mapped reads of each sample were assembled using both Scripture (beta2) [74] and Cufflinks (v2.1.1) [20] in a reference-based approach. Scripture was run with default parameters. Cufflinks was run with ‘min-frags-per-transfrag = 0’ and ‘--library-type fr-firststrand’, and other parameters were set as default. In the present study, single-exon lncRNAs were filtered out from the goat genome due to limitations of the algorithm. This operation that at least two exons are preferred is a purely technical one. To avoid false-positive results as much as possible, the transcripts with a single exon were usually considered as background transcripts and were discarded, whereas multiple-exon lncRNAs were retained [75].

### Quantification of gene expression level

Cuffdiff (v2.1.1) was used to calculate fragments per kb for a million reads (FPKM) of both lncRNAs and coding genes in each sample [20]. For biological replicates, transcripts or genes with a *P*-adjust of <0.05 were described as differentially expressed between two groups of goats with the dark and white skin.

### Coding potential and conserved analysis of lncRNAs

To achieve high-quality data, we used four analytic tools, including CNCI (v2) [76], CPC (0.9-r2) [77], Pfam-scan (v1.3) [78], and PhyloCSF (v20121028) [79] to identify the candidate lncRNAs. Transcripts predicted with coding potential by any of the four tools earlier described were filtered out, and those without coding potential were retained. Then, we selected those shared by four tools as the final candidate lncRNAs and use for further analysis. Quantification of gene expression level was estimated by calculating the FPKMs of the transcripts. The pipeline used to identify putative lncRNAs from the deep sequencing data is presented in Supplementary Figure S1.

To investigate the sequence conservation of transcripts, we used the phyloFit program in the Phast (v1.3) package [80] to compute phylogenetic models for conserved and non-conserved regions among species. Then, we used phastCons to compute a set of conservation scores of lncRNAs and coding genes.

### LncRNA target gene prediction and functional enrichment analysis

To explore the function of lncRNAs, we first predicted the target genes of lncRNAs *in cis* and *trans*. The *cis* role refers to lncRNAs’ action on neighboring target genes. In the present study, we searched coding genes 10/100 k upstream and downstream of an lncRNA. The *trans* role refers to the influence of lncRNAs on other genes at the expression level. Here, we calculated for Pearson’s correlation coefficients between expression levels of 1336 lncRNAs and 27,947 mRNAs with custom scripts (r > 0.95 or r < −0.95). Then, we performed functional enrichment analysis of the target genes for lncRNAs by using the DAVID platform [81, 82]. Significance was expressed as a *p*-value, which was calculated using the EASE score (*P* value < 0.05 was considered significant).

### Enrichment analysis of TE in goat lncRNAs

RepeatMasker (http://www.repeatmasker.org) was used with default parameters to identify various TE components in goat. To detect position bias of TEs in each class of lncRNAs, we searched for TEs at the 2000-bp upstream region of the TSS of each lncRNA identified in the goat genome (http://goat.kiz.ac.cn) and plotted its read coverage with the ggplot2 package in R [83].

### Validation of gene expression in RNA-seq by quantitative PCR analysis

Total RNAs from fetal skin in two groups of goats were used for quantitative PCR analysis. Briefly, the first cDNA strains were obtained using a One Step cDNA Synthesis Kit (Bio-Rad, USA), and were then subjected to quantification of mRNAs or lncRNAs with *β-actin* as an endogenous control using a standard SYBR Green PCR kit (Bio-Rad) on the Bio-Rad CFX96 Touch™ Real-Time PCR Detection System. The quantitative PCR was performed using the following conditions: 95 °C for 30 s, 40 cycles of 95 °C for 5 s, and the optimized annealing temperature for 30 s. The primers and annealing temperatures for 14 genes are listed in Additional file 9. All reactions were performed in triplicate for each sample. Gene expression was quantified relative to *β-actin* expression using the comparative cycle threshold (∆CT) method. Differences in gene expression between the dark and white skin were detected by using the t-test. Corrections for multiple comparisons were performed using the Holm-Sidak method.

### Statistical analysis

Data analyses were performed using the statistical R package.

### Data availability

The sequencing data were submitted to the Genome Expression Omnibus (Accession Numbers GSE69812) in NCBI.

## Additional files

Additional file 1:
**Pipeline for lncRNA analysis of the present study.** (DOCX 13 kb) Additional file 2:
**Comparison of TE components between individual subtypes of lncRNAs and mRNAs**. (XLSX 17 kb) Additional file 3:
**Comparison of TE components among three subtypes of lncRNAs.** (XLSX 13 kb) Additional file 4:
**The protein-coding genes within 10 k and 100 k upstream and downstream of an lncRNA.** (XLSX 55 kb) Additional file 5:
**Functional enrichment analysis of the protein-coding genes of lncRNAs in** ***cis.*** (XLS 360 kb) Additional file 6:
**Pearson correlations between protein-coding genes and lncRNAs.** (LIST 1829 kb) Additional file 7:
**Functional enrichment analysis of protein-coding genes of lncRNAs in** ***trans***. (XLS 7903 kb) Additional file 8:
**LncRNA-protein coding gene pairs with both co-localization and correlation relationships.** (XLS 21 kb) Additional file 9:
**Primers used in quantitative PCR analysis.** (XLSX 9 kb)

## Abbreviations

ASIP: agouti signaling protein
CREB3L1: cAMP responsive element binding protein 3-like 1
DCT: dopachrome tautomerase
ERVs: endogenous retroviruses
FZD4: frizzled-4 protein
LINEs: long interspersed elements
LncRNA: long noncoding RNA
LTRs: long terminal repeats
MITF: microphthalmia-associated transcription factor
RTEs: retrotransposable elements
SINEs: short interspersed nuclear elements
TEs: transposable elements
TSS: transcription start site
TYPR1: tyrosinase-related protein 1
