# Supplementary material for: Genome-wide analysis of long non-coding RNAs at early stage of skin pigmentation in goats (Capra hircus)
Source: BMC Genomics. 2016 Jan 19;17:67. doi: 10.1186/s12864-016-2365-3 (PMC4719336; doi:10.1186/s12864-016-2365-3)
Supplement: Additional file 1: — Pipeline for lncRNA analysis of the present study. (DOCX 13 kb) [file 12864_2016_2365_MOESM1_ESM.docx]

**RNA-seq data alignment by Tophat2**

**Assembly by Cufflinks and Scripture**

**Categorization of transcripts**

**Coding potential analysis by CPC, CNCI, pfam, and PhyloSCF**

**The candidate long non-coding RNAs**

**Characterization of subtypes of lncRNAs**

**Prediction of lncRNA function using co-expression analysis**

**Fig S1. Pipeline for analysis of skin long non-coding RNAs by RNA-sequencing.**
